# Characteristics of Renal Function in Patients Diagnosed With COVID-19: An Observational Study

**DOI:** 10.3389/fmed.2020.00409

**Published:** 2020-07-10

**Authors:** Xu-wei Hong, Ze-pai Chi, Guo-yuan Liu, Hong Huang, Shun-qi Guo, Jing-ru Fan, Xian-wei Lin, Liao-zhun Qu, Rui-lie Chen, Ling-jie Wu, Liang-yu Wang, Qi-chuan Zhang, Su-wu Wu, Ze-qun Pan, Hao Lin, Yu-hua Zhou, Yong-hai Zhang

**Affiliations:** ^1^Department of Urology, Shantou Central Hospital, Shantou, China; ^2^Department of Emergency, Shantou Central Hospital, Shantou, China; ^3^Department of Laboratory Medicine, Shantou Central Hospital, Shantou, China; ^4^Department of Nephrology, Shantou Central Hospital, Shantou, China; ^5^Department of Infectious Diseases, Shantou Central Hospital, Shantou, China; ^6^Department of Ultrasonic Medicine, Shantou Central Hospital, Shantou, China; ^7^Department of Respiratory Medicine, Shantou Central Hospital, Shantou, China; ^8^Department of Intensive Care Unit, Shantou Central Hospital, Shantou, China; ^9^Medical Department, Shantou Central Hospital, Shantou, China; ^10^Nursing Department, Shantou Central Hospital, Shantou, China

**Keywords:** 2019-nCoV, COVID-19, renal function, urinary microprotein, multiple indicators

## Abstract

**Objective:** The aim of the study was to analyze the characteristics of renal function in patients diagnosed with COVID-19.

**Methods:** In this retrospective, single-center study, we included all confirmed cases of COVID-19 in a tertiary hospital in Guangdong, China from January 20, 2020 to March 20, 2020. Blood and urine laboratory findings related to renal function were summarized, and the estimated glomerular filtration rate (eGFR) and endogenous creatinine clearance (Ccr) were also calculated to assess the renal function.

**Results:** A total of 12 admitted hospital patients were diagnosed with COVID-19, included 3 severe cases, and 9 common cases. Serum creatinine (Scr) was not abnormally elevated in all of the patients, and blood urea nitrogen (BUN) was abnormally elevated in only 25.0% of the patients. However, compared with the recovery period, the patient's Scr and BUN increased significantly in peak of disease (p-scr = 0.002 & p-bun < 0.001). By observing the fluctuations in Scr and BUN from admission to recovery, it was found that the peak of Scr and BUN appeared within the first 14 day of the course of the disease. Urinary microprotein detection indicated that the abnormally elevated rates of urine microalbumin (UMA), α1-microglobulin (A1M), urine immunoglobulin-G (IGU), and urine transferring (TRU) standardized by urinary creatinine in peak of disease were 41.7, 41.7, 50.0, and 16.7%, respectively. The abnormal rates of the calculated eGFR and Ccr were 66.7 and 41.7%.

**Conclusion:** Scr and BUN were generally increased during the course of COVID-19. Detection of urinary microproteins and application of multiple indicators assessment could be helpful for discovering abnormal renal function in patients with COVID-19. However, the evidence is limited due to the small sample size and observational nature. Additional studies, especially large prospective cohort studies, are required to confirm these findings.

## Introduction

Since December 2019, unexplained clustered pneumonia cases have started to appear in Wuhan, Hubei Province, China. It is identified as a pneumonia caused by a novel coronavirus infection (1, 2). The World Health Organization (WHO) named the coronavirus 2019-nCoV, which was the third coronavirus that infects humans from wild hosts across germlines after severe acute respiratory syndrome coronavirus (SARS-CoV) and Middle East respiratory syndrome coronavirus (MERS-CoV) and can cause severe pneumonia. It is also the seventh species of pathogenic human respiratory coronaviruses (3, 4). On February 11, 2020, the WHO named the novel coronavirus-infected disease2019 novel coronavirus disease shorted for COVID-19. With the spread of the epidemic, other cases in China and abroad have also occurred (5). The spread of disease has caused huge lives and economic losses to the world.

The 2019-nCoV human infection cases combine the characteristics of the previous 6 coronaviruses, which can cause mild cases, easily lose vigilance, and can also cause severe cases, with a high mortality rate (6). Multiple organ failure is the main cause of death of COVID-19. The latest research reports that the incidence of COVID-19 with organ dysfunction is about 33%, of which the acute renal injury is about 3~7% (7, 8). Impaired renal function can lead to obstruction of excretion of metabolites and toxins in the body, which will adversely affect the maintenance of the electrolyte and acid-base balance of the human body. In addition, when renal function is severely damaged, uremia will occur, and endanger life. Early detection of evidence of renal injury and timely effective interventions are of great significance for reducing complications and improving prognosis.

This study intends to use a number of laboratory test indexes, including serum creatinine, blood urea nitrogen, urine creatinine, urine microprotein, and eGFR et al. to comprehensively assess renal function and make a deep characterization of renal function in patients diagnosed with COVID-19.

## Materials and Methods

### Patients

From January 20, 2020 to March 20, 2020, 12 patients with pneumonia of unknown cause were admitted to a tertiary hospital in Guangdong, China. Nasopharyngeal swab samples of all patients were tested positive for 2019-nCoV virus nucleic acid and confirmed to be infected with 2019-nCoV by Guangdong Province CDC (Center for Disease Control and Prevention). The disease diagnostic criteria and case classification were refer to the guidelines for diagnosis and treatment of COVID-19 (Trial Version 7) issued by China National Health Commission (9).

### Data Collection

A standardized case collection form was designed to collect case data. It mainly included the following information: (1) General information: gender, age, weight. (2) Medical history: fever, chills, sore throat, cough, headache, fatigue, myalgia, diarrhea, and other symptoms, hypertension, diabetes, chronic lung, heart, kidney, liver disease history, and history of malignant tumor. (3) Laboratory data: blood routine, electrolytes, liver and kidney function, myocardial enzymes, heart failure indicators, coagulation function, infection indexes, etc. Urine routine, urine creatinine, urinary microprotein detection, etc.

### Evaluation of Renal Function

Serum creatinine (Scr), blood urea nitrogen (BUN), urine protein (PRO), urine occult blood urine (BLD), 24-h urine creatinine (UCr), 24-h urine K (UK), 24-h urine Na (UNa), microalbumin (UMA), α1-microglobulin (A1M), urine immunoglobulin G (IGU), and urine transferrin (TRU) were detected. The estimated glomerular filtration rate (eGFR) and endogenous creatinine clearance (Ccr) were calculated.

Among them, the eGFR was calculated according to the simplified MDRD

formula modified by the Chinese.

$$\begin{array}{l} \text{eGFR}(\text{ml}/\left({\text{min}}^{*}1.73{\text{m}}^{2}\right)=186\times {\text{Scr}\left(\text{mg}/\text{dl}\right)}^{-}1.154\times \text{Age}\\ \;{\left(\text{year}-\text{old}\right)}^{-}0.203\left(\text{female}\times 0.742\right). \end{array}$$

(the Counahan-Barrat method was applied when the patient is younger than 14- year-old)

The Ccr was calculated according to Cockcroft's formula.

$$\begin{array}{l} \text{Ccr}=\left[140-\text{Age}\left(\text{year}-\text{old}\right)\right]\times \text{weight}\left(\text{kg}\right)÷\left[72\times \text{Scr}\left(\text{mg}/\text{dl}\right)\right]\\ \;\left(\text{female}\times 0.85\right). \end{array}$$

### Statistical Methods

The paired-samples *T*-test was used to compare the differences of Scr and BUN between patients in the course of disease and in the period of recovery. It was considered statistically significant when the *P*-value was < 0.05. All statistical analyses were processed using SPSS 25.0 statistical software.

## Results

### Clinical Characteristics of the COVID-19 Patients

Of the 12 patients with COVID-19, 3 (25.0%) and 9 (75.0%) were categorized into severe and common group, respectively. The severe patients required oxygen to support, the average oxygen saturation (SpO2) of the severe patients was 86.7% (81–92%). The common group included 7 (58.3%) mild cases and 2 (16.7%) non-pneumonia cases. There were 7 (58.3%) male and 5 (41.7%) female patients. The oldest patient was 69 years old and the youngest was 12 years old, with an average age of 41.3 ± 18.2 years old. Among the patients, only one patient (case 9) had never been to Wuhan City and became ill after contacting a person diagnosed with COVID-19 after going on a trip. The remaining 11 patients all came from Wuhan city within 14 days. Clinical symptoms of 12 patients included fever, sore throat, cough, headache, fatigue, myalgia, and some patients had no obvious clinical symptoms. Three patients had pre-existing diseases, including diabetes, hypertension, and chronic emphysema, as shown in Table S1.

### Laboratory Characteristics of the COVID-19 Patients

Laboratory testing items included blood routine, electrolyte, metabolism, heart, liver, kidney function indicators, coagulation function, and infection indicators. As shown in Table S2, the common abnormal indicators (abnormal rate ≥ 50%) included: increased neutrophil ratio (50%), increased monocyte ratio (75%), hypokalemia (50%), hyponatremia (50%), hypoproteinemia (75%), and increased C-reactive protein (83.3%).

### Characteristics of Renal Function of the COVID-19 Patients

**Figure 1:**
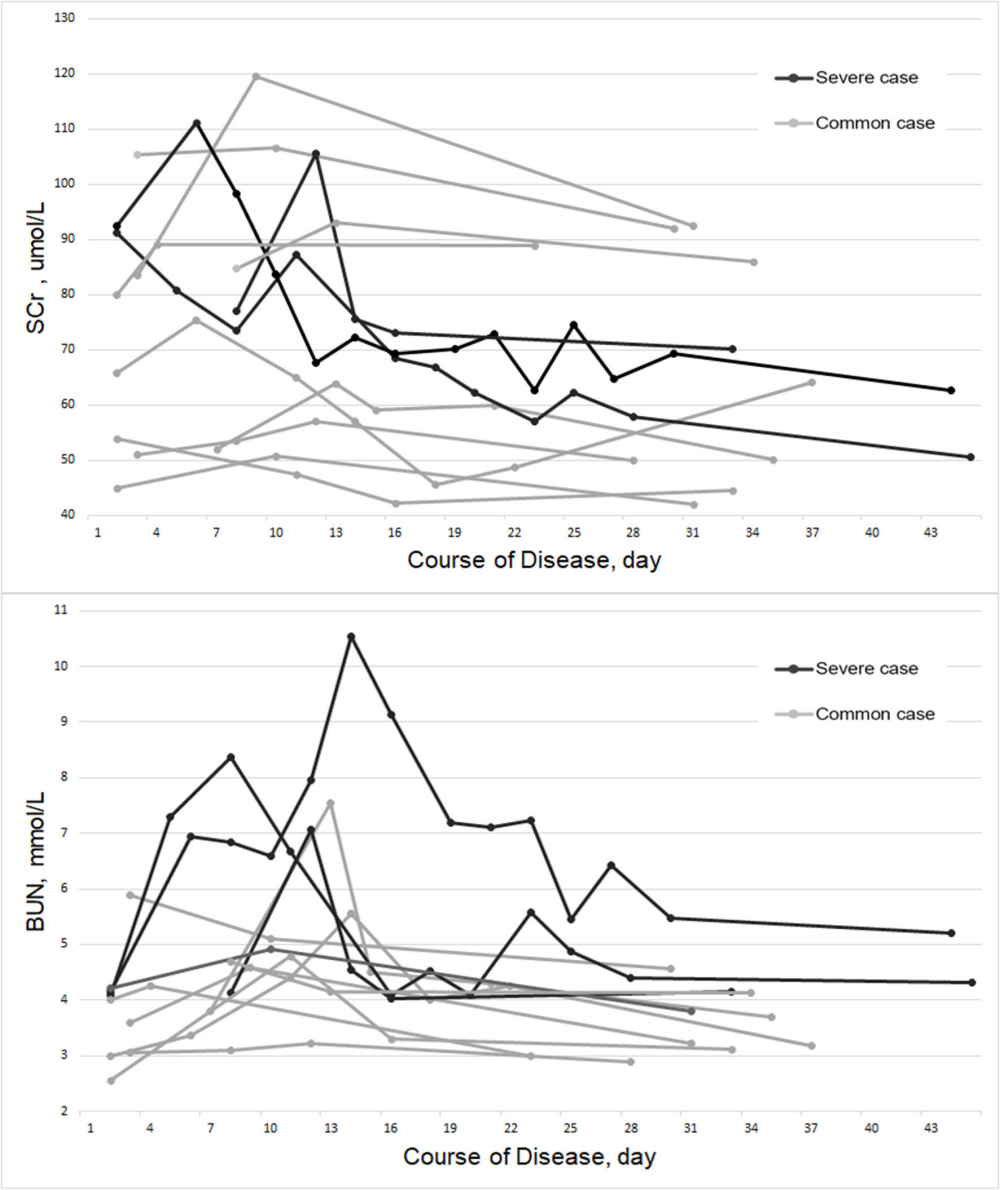
Fluctuations in Scr and BUN of the COVID-19 patients from admission to recovery.

As shown in Table 1, Scr was not abnormally elevated in all of the patients, and BUN was abnormally elevated in only 25.0% of the patients. However, compared with the recovery period, which was defined as the virus nucleic acid test of 2019-nCoV turned negative and at least 2 weeks after discharge, the patient's Scr and BUN increased significantly in peak of disease (p-scr = 0.002 & p-bun < 0.001). By conducting subgroup analyses, stratified by case classification (severe vs. common case), gender (male vs. female) and age (≥50 and <50 years old), the results were found to be consistent with the primary findings (Table 2). By observing the fluctuations in Scr and BUN from admission to recovery, we found that the peak values appeared within the first 14 days of the course of the disease, as shown in Figure 1.

**Table 1:** Renal function indicators of the COVID-19 patients in peak of disease.

**Renal function indexes**	**Normal range**	**Case 1**	**Case 2**	**Case 3**	**Case 4**	**Case 5**	**Case 6**	**Case 7**	**Case 8**	**Case 9**	**Case 10**	**Case 11**	**Case 12**	**Abnormal rate (%)**
**Blood detection**														
Scr, umol/L	40~133	63.8	75.3	119.5	106.6	91.3	111.2	105.5	50.8	53.8	89.1	93.1	56.9	0
BUN, mmol/L	2.5~7.14	7.54	5.55	4.59	5.88	8.37	10.54	7.07	4.91	4.80	4.25	4.69	3.22	25.0
eGFR, ml/(min*1.73 m2)	≥90	85.9	75.7	66.8	87.7	75.9	62.6	67.7	112	105.4	91.8	84.2	110.3	66.7
Ccr, ml/min	≥80	78.3	70.9	83.6	100.6	71.3	63.1	78.4	104.7	93.4	82.2	104.1	143.0	41.7
**Urine detection**														
PRO	–	–	–	1+	–	2+	2+	1+	–	1+	–	–	–	41.7
BLD	–	–	1+	–	–	–	–	–	–	–	–	–	–	8.3
24H UCr, umol/24 h	7,000–17,600	9,126	9,666	12,428	NA	9,760	15,369	11,478	NA	11,436	NA	11,077	7,084	0
24H UK, mmol/24 h	25–100	156	85	56	NA	125	168	224	NA	27	NA	25	36	44.4
24H UNa, mmol/24 h	130–260	206	311	145	NA	320	360	282	NA	363	NA	111	104	55.6
**Urinary micro-protein detection**														
UMA/Ucr, mg/g	<30	11.3	9.5	35.2	7.07	58	60.5	42.9	10.1	32.5	6.4	22.2	5.8	41.7
A1M/Ucr, mg/g	<20	12.4	54.8	10.6	4.7	133.4	123.2	107.7	10.5	29.4	18.2	16.7	16.2	41.7
IGU/Ucr, mg/g	<12.6	8.8	14.3	5.6	5.7	20.7	64.6	43.9	9.6	34.0	7.7	14.2	9.4	50.0
TRU/Ucr, mg/g	<3.2	3.0	9.5	2.8	2.1	3.5	4.1	9.1	2.8	3.0	2.5	2.8	3.0	33.3

*Scr, serum creatinine; BUN, blood urea nitrogen; eGFR, estimated glomerular filtration rate; Ccr, endogenous creatinine clearance; PRO, urine protein; BLD, urine occult blood; Ucr, urine creatinine; UK, urine K; UNa, urine Na; UMA, urine microalbumin; A1M, α1-microglobulin; IGU, urine immunoglobulin G; TRU, urine transferrin; NA, no accepted*.

**Table 2:** Comparison of Scr and BUN between peak of disease and recovery period.

		**Scr (umol/L)**			**BUN (mmol/L)**		
	**Cases**	**Peak of disease**	**Recovery period**	***P***	**Peak of disease**	**Recovery period**	***P***
Total	12	84.74 ± 24.05	66.14 ± 19.36	0.002	5.95 ± 2.07	3.77 ± 0.72	<0.001
Case classification							
Severe	3	102.67 ± 10.25	61.13 ± 9.94	0.008	8.66 ± 1.75	4.56 ± 0.56	0.028
Common	9	78.77 ± 24.66	67.81 ± 21.87	0.002	5.04 ± 1.20	3.51 ± 0.57	0.002
Gender							
Male	7	102.33 ± 11.43	77.56 ± 16.54	0.011	6.48 ± 2.32	4.08 ± 0.76	0.011
Female	5	60.12 ± 9.76	50.16 ± 8.56	0.001	5.20 ± 1.56	3.33 ± 0.39	0.035
Age							
≥50	4	92.95 ± 21.16	58.40 ± 9.79	0.019	8.38 ± 1.54	4.34 ± 0.63	0.004
<50	8	80.64 ± 25.67	70.01 ± 22.28	0.006	4.74 ± 0.81	3.49 ± 0.61	0.001

*Scr, Serum creatinine; BUN, Blood urea nitrogen*.

Urinary microprotein detection showed that the abnormally elevated rates of urine microalbumin (UMA), α1-microglobulin (A1M), urine immunoglobulin-G (IGU), and urine transferring (TRU) standardized by urinary creatinine in peak of disease were 41.7, 41.7, 50.0, and 16.7%, respectively. The abnormal rates of calculated eGFR and Ccr were 66.7 and 41.7% (Table 1).

## Discussion

Although diffuse alveolar damage and acute respiratory failure are the main features of COVID-19, the involvement of other organs also need to be considered. Early data suggests that acute kidney impairment is more common in COVID-19 patients than in patients with other coronavirus syndromes, and that kidney impairment associates with mortality (10). However, very little is known about renal involvement in COVID-19 patients. The present study intends to summarize the renal laboratory findings and make a deep characterization of renal function in COVID-19.

In this study, we report the renal laboratory findings in 12 sequential COVID-19 patients. The abnormally elevated rates of Scr and BUN are quite low. However, compared with the recovery period, the patient's Scr and BUN increased significantly in peak of disease. The abnormal rates of the calculated eGFR and Ccr are also higher in peak of disease. Scr and BUN are commonly used indicators for the detection of renal function. However, the sensitivities of both of them are poor. Usually, abnormalities occur only when renal function is significantly damaged. By calculating eGFR and CCr, which relies on age, gender, weight, and Scr, it can better reflect the early renal function impairment. The microproteins in urine are potential early biomarkers associated with renal impairment, most of them are closely related to glomerular filtration function and renal reabsorption function. Urinary microprotein detection in the included 12 COVID-19 patients find that the abnormally elevated rates of UMA, A1M, IGU, and TRU standardized by urinary creatinine in peak of disease are higher. The study also find that hypokalemia and hyponatremia are common in patients with COVID-19. By conducting 24-h urine electrolyte testing, it is found that patients with hypokalemia and hyponatremia also have excessive potassium and urinary sodium excretion, which suggest that these patients may have renal tubular reabsorption dysfunction.

Previously, an ongoing case study reported 59 patients infected by 2019-nCoV, including 28 severe cases and 3 death. In that study, 63% of the patients exhibited proteinuria, and 19 and 27% of the patients had an elevated level of Scr and BUN, respectively. Computed tomography (CT) scans revealed abnormal renal imaging in all patients. The results suggest that renal impairment is common in COVID-19, which may contribute to multiorgan failure and death eventually (11). Zhou et al. (12) reported the identification and characterization of 2019-nCoV. Through full-length genome sequences analysis, they found that the whole genome of 2019-nCoV shared 79.5% sequence identify to SARS-CoV. The pairwise protein sequence analysis of seven conserved non-structural proteins showed that this virus belongs to the species of severe acute respirator syndrome-related coronaviruses (SARSr-CoV). In addition, they also confirmed that 2019-nCov used the same cell entry receptor, Angiotensin converting enzyme II (ACE2), as SARS-CoV. The high degree of similarity in gene sequence and cellular mechanism of 2019-nCoV and SARS-CoV suggests that the risk factors of mortality could also be similar (12). Zou et al. (13) analyzed the single-cell RNA sequencing datasets to explore the expression of ACE2 in the main physiological systems of the human body, including the respiratory, cardiovascular, digestive and urinary system. The study showed that heart, esophagus, kidney, bladder, and ileum have similar or higher ACE2 expression than in alveoli. In the analysis of specific cell types, the expression of ACE2 in renal proximal tubule cells was about four times higher than that of type II alveolar cells (AT2) (13). The results suggest that the kidney may be one of the primary targets of attack for the 2019-nCov.

### Strengths and Limitations

This is an initial study on renal function associated with COVID-19. It is important to show the clinical evidence of infection of 2019-nCoV which is not simply pneumonia. In this study, we summarize the renal laboratory findings in 12 sequential COVID-19 patients, addressing a critical question with data which is not available from other sources. This summary of data is important to share with the scientific community. However, the present study has some limitations that must be taken into account when considering its contribution. First and most significantly is the small sample size of this study. COVID-19 is a newly emerging epidemic. The cases found in this city are all imported cases. After strict prevention and control, there are fewer confirmed cases in this city. Therefore, the number of cases included in this research is also small. Secondly, considering COVID-19 is a completely new disease, and we do not yet fully know the characteristics of its cases, the type of research used in this study is designed as a descriptive study, which initially explores the basic characteristics of the disease and does not set the control group. Thirdly, in this study, we have evaluated some potential early biomarkers of kidney injury, such as UMA, IGU, and TRU. However, there are still some important biomarkers that have not been detected, like urinary Kim-1, MCP-1, and NGAL. As a retrospective study, we cannot retest these indicators. For the same reason, we are unable to retest the urine samples of patients to see if the 2019-nCoV could be detected. If there are new cases, we will try to tested the urine 2019-nCoV, and we hope our study can inspire more researchers to carry out relevant studies.

## Conclusions

This study found that Scr and BUN were generally increased during the course of COVID-19. Detection of urinary microproteins and application of multiple indicators assessment could be helpful for discovering abnormal renal function in patients with COVID-19. However, the evidence is limited due to the small sample size and observational nature. Additional studies, especially large prospective cohort studies, are required to confirm these findings.

## Data Availability Statement

All datasets generated for this study are included in the article/Supplementary Material.

## Ethics Statement

The studies involving human participants were reviewed and approved by the institutional research ethics committee of Shantou Central Hospital. Written informed consent to participate in this study was provided by the participants.

## Author Contributions

YZha obtained funding. XH and LQ designed the study. HH, JF, XL, LWu, and YZho collected the data. ZC and GL were involved in data verification and analyzed the data. SG, RC, QZ, SW, LWa, ZP, and HL are responsible for the diagnosis and treatment of patients. XH drafted the manuscript. YZha and LQ contributed to the interpretation of the results and critical revision of the manuscript for important intellectual content and approved the final version of the manuscript. All authors have read and approved the final manuscript.

## Conflict of Interest

The authors declare that the research was conducted in the absence of any commercial or financial relationships that could be construed as a potential conflict of interest.
