# Supplementary material for: Characteristics of Renal Function in Patients Diagnosed With COVID-19: An Observational Study
Source: Front Med (Lausanne). 2020 Jul 10;7:409. doi: 10.3389/fmed.2020.00409 (PMC7365839; doi:10.3389/fmed.2020.00409)
Supplement: Supplementary file 1 [file Table_1.DOCX]

| **Supplementary tables**  **Table S1.** Clinical characteristics of the COVID-19 patients | | | | | | | | | | | | |
| --- | --- | --- | --- | --- | --- | --- | --- | --- | --- | --- | --- | --- |
| **Characteristics** | **Case 1** | **Case 2** | **Case 3** | **Case 4** | **Case 5** | **Case 6** | **Case 7** | **Case 8** | **Case 9** | **Case 10** | **Case 11** | **Case 12** |
| Gender | Female | Female | Male | Male | Male | Male | Male | Female | Female | Male | Male | Female |
| Age | 66 | 33 | 28 | 14 | 69 | 58 | 53 | 12 | 47 | 31 | 37 | 37 |
| Wuhan sojourn history | Yes | Yes | Yes | Yes | Yes | Yes | Yes | Yes | No | Yes | Yes | Yes |
| **Initial symptoms** |  |  |  |  |  |  |  |  |  |  |  |  |
| Fever | No | No | Yes | Yes | No | Yes | Yes | No | No | Yes | No | No |
| Chill | No | No | No | No | No | No | No | No | No | No | No | No |
| Sore throat | Yes | Yes | Yes | No | No | Yes | No | No | Yes | No | Yes | No |
| Cough | Yes | No | Yes | No | No | Yes | No | Yes | Yes | No | No | No |
| Headache | No | No | Yes | No | No | No | Yes | No | No | No | No | Yes |
| Fatigue | Yes | No | Yes | No | No | No | No | No | No | No | No | No |
| Myalgia | Yes | No | No | No | No | No | No | No | No | No | No | No |
| Diarrhea | No | No | No | No | No | No | No | No | No | No | No | No |
| Pneumonia (CT/X-ray) | Yes | Yes | Yes | Yes | Yes | Yes | Yes | No | Yes | No | Yes | Yes |
| **Pre-existing conditions** |  |  |  |  |  |  |  |  |  |  |  |  |
| Hypertension | No | No | No | No | No | No | Yes | No | No | No | No | No |
| Diabetes | No | Yes | No | No | Yes | No | No | No | No | No | No | No |
| Chronic lung disease | No | No | No | No | Yes | No | Yes | No | No | No | No | No |
| Chronic heart disease | No | No | No | No | No | No | No | No | No | No | No | No |
| Chronic kidney disease | No | No | No | No | No | No | No | No | No | No | No | No |
| Chronic liver disease | No | No | No | No | No | No | No | No | No | No | No | No |
| Malignant tumor | No | No | No | No | No | No | No | No | No | No | No | No |
|  |  |  |  |  |  |  |  |  |  |  |  |  |
| **Case classification** | common | common | common | common | severe | severe | severe | common | common | common | common | common |
