# Supplementary material for: Characteristics of Renal Function in Patients Diagnosed With COVID-19: An Observational Study
Source: Front Med (Lausanne). 2020 Jul 10;7:409. doi: 10.3389/fmed.2020.00409 (PMC7365839; doi:10.3389/fmed.2020.00409)
Supplement: Supplementary file 2 [file Table_2.DOCX]

| **Supplementary tables**  **Table 2.** Laboratory test results of the COVID-19 patients at admission | | | | | | | | | | | | | | | | | | |
| --- | --- | --- | --- | --- | --- | --- | --- | --- | --- | --- | --- | --- | --- | --- | --- | --- | --- | --- |
| **Laboratory test results** | **Normal range** | **Case 1** | **Case 2** | | **Case 3** | **Case 4** | | | | **Case 5** | **Case 6** | **Case 7** | **Case 8** | **Case 9** | **Case 10** | **Case 11** | **Case 12** | **Abnormal rate (%)** |
| **Blood routine** |  |  | |  |  |  | | | |  |  |  |  |  |  |  |  |  |
| White blood cell (WBC，10^9/L) | 3.5~9.5 | 11.4 | 12.6 | | 6.3 | 4.8 | | | | 7.4 | 16.2 | 3.7 | 4.5 | 5.7 | 6 | 5.5 | 4.1 | 25 |
| Neutrophil ratio (NER，%) | 50~70 | 89.6 | 78.4 | | 51 | 48.8 | | | | 74.3 | 92.6 | 61 | 27.9 | 59.6 | 58.2 | 53.6 | 50.1 | 50 |
| Lymphocyte ratio (LYR，%) | 20~40 | 8.3 | 15.1 | | 36 | 38.4 | | | | 17.1 | 1.3 | 24 | 60 | 27.5 | 30.3 | 27.4 | 39.2 | 33.3 |
| Monocyte ratio (MOR，%) | 3~8 | 2 | 4.9 | | 11.6 | 10.7 | | | | 7.1 | 6 | 14.2 | 8.6 | 11.7 | 8.6 | 16.1 | 8.8 | 75 |
| Platelet (PLT，10^9/L) | 125~350 | 310 | 246 | | 367 | 254 | | | | 268 | 133 | 117 | 239 | 273 | 288 | 247 | 188 | 16.7 |
| Hemoglobin (HGB，g/L) | 130~175(male)  115~150(female) | 107 | 132 | | 161 | 156 | | | | 129 | 124 | 162 | 122 | 101 | 143 | 153 | 127 | 33.3 |
| **Biochemical indexes of kidney, liver, and heart function** | | | | | |  |  |  |  | |  |  |  |  |  |  |  |  |
| Serum K (K，mmol/L) | 3.5~5.3 | 3.09 | 3.48 | | 3.26 | 4.03 | | | | 3.74 | 3.14 | 3.05 | 3.84 | 3.46 | 4.48 | 3.89 | 3.91 | 50 |
| Serum Na（Na，mmol/L） | 137~147 | 135 | 131 | | 138.7 | 139 | | | | 131 | 136 | 136 | 138 | 132 | 139 | 139 | 137 | 50 |
| Glucose (GLU，mmol/L) | 3.89~6.11 | 5.04 | 10.78 | | 5.01 | 4.43 | | | | 6.15 | 9.51 | 5.16 | 4.69 | 5.13 | 4.93 | 5.34 | 5.13 | 25 |
| Blood urea nitrogen (BUN，mmol/L) | 2.5~7.14 | 3.80 | 3.00 | | 3.60 | 5.88 | | | | 4.09 | 4.15 | 4.13 | 4.21 | 2.55 | 4.00 | 4.69 | 3.05 | 0 |
| Serum creatinine (Scr，umol/L) | 40~133 | 52.1 | 65.7 | | 83.5 | 105.3 | | | | 91.3 | 92.5 | 77.0 | 44.9 | 53.8 | 80.0 | 84.7 | 51.0 | 0 |
| Uric acid (UA，umol/L) | 150~430 | 220 | 206 | | 465 | 356 | | | | 305 | 266 | 468 | 331 | 202 | 284 | 446 | 194 | 25 |
| Albumin (ALB，g/L) | 40~55 | 33.7 | 34.6 | | 42.8 | 46.2 | | | | 29.4 | 36 | 34.3 | 36.7 | 39.4 | 43.6 | 39.6 | 35 | 75 |
| Triglyceride (TG，mmol/L) | 0.23-1.71 | 1.05 | 0.95 | | 2.43 | 2.22 | | | | 1.7 | 1.07 | 2.58 | 1.3 | 1.37 | 0.97 | 3.3 | 2.15 | 41.7 |
| Cholesterol (CH，mmol/L) | 2.9~5.72 | 5.2 | 3.61 | | 4.23 | 4.55 | | | | 5.1 | 4.43 | 4.05 | 5.65 | 4.86 | 4.67 | 4.34 | 4.44 | 0 |
| Total bilirubin (TBIL，umol/L) | 2~20 | 16.1 | 25.4 | | 30.6 | 16.9 | | | | 19.3 | 13.7 | 13 | 19 | 13.6 | 14.7 | 15.7 | 14.1 | 16.7 |
| Glutamic pyruvic transaminase (ALT，U/L) | 9~50(male) 7~40(female) | 22 | 10 | | 34 | 12 | | | | 19 | 21 | 19 | 16 | 12 | 23 | 31 | 16 | 0 |
| Glutamic oxaloacetic transaminase (AST，U/L) | 15~40(male) 13~35(female) | 32 | 14 | | 23 | 18 | | | | 21 | 33 | 17 | 28 | 23 | 22 | 21 | 15 | 0 |
| Creatine kinase (CK，U/L) | 0~174 | 79 | 69 | | 71 | 76 | | | | 34 | 373 | 26 | 97 | 58 | 68 | 69 | 35 | 8.3 |
| Lactic dehydrogenase (LDH，U/L) | 100~300 | 332 | 194 | | 226 | 193 | | | | 171 | 270 | 199 | 175 | 220 | 149 | 181 | 202 | 8.3 |
| Troponin I (aTnI，ng/Ml) | 0~0.04 | 0.001 | 0 | | 0 | 0 | | | | 0 | 0.03 | 0 | 0 | 0 | 0 | 0 | 0 | 0 |
| B-type natriuretic peptide (BNP，pg/mL) | 0~300 | 220 | 60 | | 120 | 30 | | | | 205.3 | 556.2 | 280 | 20 | 125 | 224 | 60.7 | <18 | 8.3 |
| **Coagulation function** |  |  |  | |  |  | | | |  |  |  |  |  |  |  |  |  |
| Fibrinogen (FIB-C，g/L) | 1.8~4.88 | 5.42 | 2.3 | | 2.5 | 3.2 | | | | 8.4 | 5.88 | 4.85 | 2.5 | 3.49 | 1.53 | 2.9 | 2.64 | 25 |
| Plasma d-dimer (D-Dimer，ug/L) | 0~550 | 310 | 150 | | 164 | 220 | | | | 1920 | 1470 | 290 | 153 | 2110 | 750 | 320 | 160 | 33.3 |
| **Infection indexes** |  |  |  | |  |  | | | |  |  |  |  |  |  |  |  |  |
| C-reaction protein (CRP，mg/L) | 0~8 | 74.7 | 29.06 | | 19.6 | 5.2 | | | | 113.76 | 124.78 | 33.43 | <0.499 | 15.3 | 1.14 | 5.61 | 2.99 | 83.3 |
| Procalcitonin (PCT，ng/mL) | 0~0.5 | 0.05 | 0.034 | | <0.1 | <0.1 | | | | 0.112 | 0.116 | <0.1 | <0.02 | <0.1 | <0.1 | 0.13 | <0.1 | 16.7 |
